# New insights in amino sugar metabolism by the gut microbiome

**DOI:** 10.1080/19490976.2025.2510462

**Published:** 2025-05-25

**Authors:** Wenqiang Shen, Jan Freark de Boer, Folkert Kuipers, Jingyuan Fu

**Affiliations:** a Department of Pediatrics, University of Groningen, University Medical Center Groningen, Groningen, The Netherlands; b Department of Laboratory Medicine, University of Groningen, University Medical Center Groningen, Groningen, The Netherlands; c European Research Institute for the Biology of Ageing (ERIBA), University of Groningen, University Medical Center Groningen, Groningen, The Netherlands; d Department of Genetics, University of Groningen, University Medical Center Groningen, Groningen, The Netherlands

**Keywords:** Gut microbiome, amino sugar metabolism, GalNAc utilization pathway, host health

## Abstract

Gut microorganisms inhabiting the intestinal tract play key roles in host’s health and disease. A properly functioning gut microbiome requires the availability of adequate carbon, nitrogen and energy sources. One of the main sources of energy for intestinal bacteria are glycans, of which amino sugars are important components. Amino sugars are a class of carbohydrates in which one or more hydroxyl groups are substituted with amino groups. However, bacterial utilization of amino sugars and their impact on the gut microbiome and host health have not been thoroughly assessed. In this review, we summarize the latest discoveries about amino sugar metabolism by gut microbes, paying particular attention to the metabolism of N-acetyl-galactosamine (GalNAc), one of the most abundant amino sugars in the intestine, and its potential implications for microbial functionality and host health.

The gastrointestinal tract contains tens of trillions of microorganisms, collectively known as the gut microbiota. Its composition is regulated by host genetics,^1^ environmental factors and diet composition, and by interactions between these factors.^2^ Given the large metabolic capacity of the gut microbiota and the impact of microbiota-derived metabolites on host health, the gut microbiota is now considered an important metabolic ‘organ’.^3–5^ Maintaining or reestablishing a healthy microbiota has therefore become a viable strategy for improving host health(span). Maintenance of a ‘healthy microbiota’ requires the availability of adequate sources of energy, carbon and nitrogen. In this context, glycans are major sources of nutrition for intestinal bacteria, and these molecules have been shown to play important roles in regulating microbial composition and host (patho)physiology.^6^

Glycans are simple or complex carbohydrates that can be present in the intestinal lumen in the form of free sugars, small-molecule oligosaccharides, polymers of multiple monosaccharides, or polymers connected to other biomolecules by covalent linkages, such as proteins (glycoproteins), peptides (glycopeptides), lipids (glycolipids)^7^ and even small noncoding RNAs (glycoRNAs).^8^ In contrast to accumulating homopolysaccharides such as glycogen and amylose, the sugar moieties of glycoconjugates are generally complex heteropolysaccharides. The chemical structure of glycans can vary greatly, depending on their sources (Figure 1).

**Figure 1.**
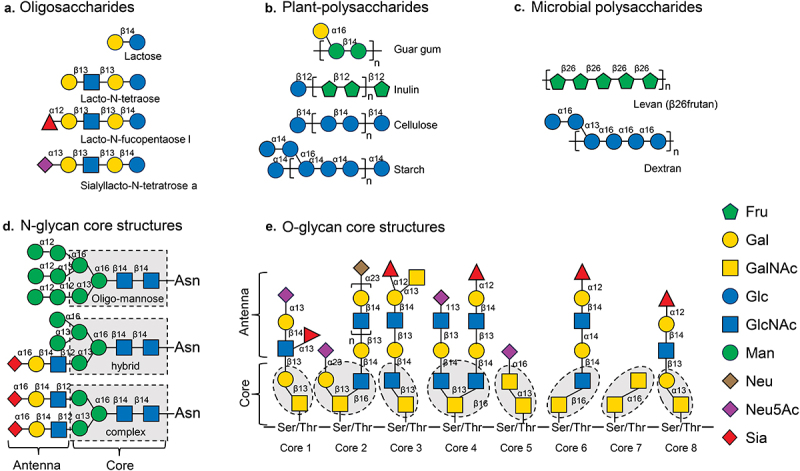
The chemical structure of glycans from different sources. Panels show the schematic of each monosaccharide using symbol nomenclature for glycans format. (a) oligosaccharides. (b) plant-derived polysaccharides. (c) microbial polysaccharides. (d) structures of N-linked glycans, which generally contain three types: oligo-mannose, hybrid and complex. Each N-glycan contains the common core Man3GlcNAc2Asn (gray rectangle) but with different extensions (antenna). (e) structures of O-linked glycans, which have eight extensible core structures (gray oval) with additional sugar extensions (antenna). As implied in their names, the GlcNAc moiety in the core structure of N-linked glycans binds to the amide nitrogen (N) atom of an asparagine (Asn) residue, while the GalNAc moiety in the core structure of O-linked glycans commonly binds to an oxygen (O) atom of serine (ser) or threonine (Thr) residues. Fru: Fructose; Gal: Galactose; GalNAc: N-acetyl-galactosamine; Glc: Glucose; GlcNAc: N-acetyl-glucosamine; man: Mannose; Neu: neuraminic acid; Neu5Ac: N-acetyl-neuraminic acid; Sia: sialic acids.

Monosaccharides are the simplest glycans and comprise molecules such as glucose, mannose, galactose and N-acetyl-galactosamine (GalNAc). A disaccharide is formed when a hydroxyl group of one monosaccharide reacts with the hemiacetal/hemiketal group of another to form an acetal/ketal moiety, while oligosaccharides usually contain between two and a dozen monosaccharide residues. The larger structures are usually referred to as polysaccharides, of which glycogen, starch, cellulose and dietary fibers are common examples (Figure 1). As mentioned above, these carbohydrates can be covalently attached to proteins or lipids to form glycoconjugates. These co-translational or post-translational modifications, referred to as glycosylation, are of pivotal importance for the structure and function of glycan-containing molecules.^9^

There are two main kinds of glycoconjugates according to glycosylation location, namely nitrogen- linked (N-linked) and oxygen-linked (O-linked) glycans. Several less common types of glycosylation also exist, such as carbon (C-), phospho (*p*-) and sulfur (S)-glycosylation.

## Glycans are the major energy sources for the gut microbiota

**Figure 2.**
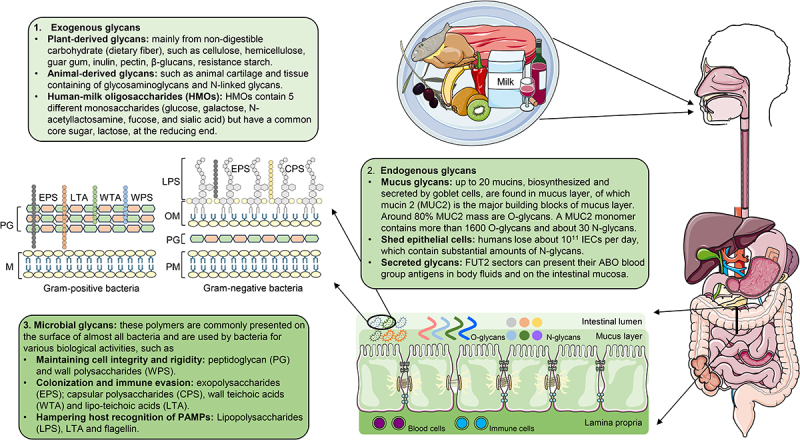
Sources of glycans accessed and utilized by gut microbes. These can be classified into three categories according to their origin: 1. exogenous glycans, 2. endogenous glycans and 3. microbial glycans. If the host diet provides sufficient exogenous glycans, gut microbiota can use them as main energy sources, maintaining a balanced health status. However, if the host diet is short of exogenous glycans, e.g., dietary fiber, bacteria will turn to utilizing glycans from the mucus layer to get energy, causing damage to the intestine. Animal-derived glycans, and in infants human milk oligosaccharides, are seen as prebiotics because they have similar structures to O-glycans in mucins. IECs, intestinal epithelial cells; PAMPs, pathogen-associated molecular patterns.

Glycans play crucial roles in maintaining gut health.^10–12^ Based on their sources, they can be classified as exogenous, endogenous or microbial glycans (Figure 2).^6^

Quantitatively, the main influx of glycans into the intestine comes from exogenous glycans derived from the host diet, a source that contains plant-based and animal-derived glycans, as well as human milk oligosaccharides (HMOs) in newborns (Figure 2). These glycans represent the predominant energy source for the gut microbiota. Plant-based glycans usually contain large amounts of non-digestible forms of carbohydrates, i.e., dietary fibers. As digestible carbohydrates such as starch barely reach the microbiota in the distal intestine, we do not discuss them in this review. Polysaccharides from plant cell walls are either non-fermentable (e.g., cellulose and methylcellulose) or poorly-fermentable (e.g., hemicellulose) by human gut bacteria, but they aid in defecation due to their hydrophilic bulking properties. On the other hand, most polysaccharides (guar gum and inulin, Figure 1) are highly fermentable by gut bacteria, producing short-chain fatty acids (SCFAs), which have a range of health benefits for the host and serve as an energy supply for intestinal epithelial cells (IECs), especially colonocytes [see Box].^13^ Animal-derived glycans often consist of *N*- or O-glycosylated peptides and proteins,^14^ such as the glycosaminoglycans and N-linked glycans enriched in animal cartilage and tissues. HMOs are oligosaccharides that are abundant in human breast milk, with ~200 types of HMOs identified to date. HMOs play a multifaceted role in supporting infant health and development since they act as prebiotics by stimulating colonization of the infant’s intestine by beneficial microbes (mainly *Bifidobacterium*) and protect against enteric pathogens [see Box].^15^

Endogenous glycans present in the intestinal lumen primarily originate from secreted mucus glycoproteins (O-linked) and shed IECs (N-linked). These glycans are localized in the mucus layer that covers the intestinal wall. Currently, ~20 mucin glycans have been identified in humans.^24^ The main component of the intestinal mucus layer is mucin 2 (MUC2), with about 80% of its molecular mass coming from O-glycans.^25^ Human IECs have a high turnover rate and can self-renew within 3–5 days. This rapid turnover, together with persistent invasion from luminal contents, results in the loss of more than 10^11^ epithelial cells (~200 g) per day in humans,^26,27^ providing the intestinal flora with a substantial amount of N-linked glycans. Compared to exogenous glycans, which show wide diversity and large fluctuations in composition and abundance, endogenous glycans are more stable and consistent nutrient sources for bacteria. Additionally, in individuals classified as *FUT2* (the gene encoding fucosyltransferase 2) secretors, glycans of their ABO blood group antigens can be secreted into the gastrointestinal mucosa and hence be encountered by gut bacteria.^28,29^ Consequently, these glycans can serve as an alternative energy source for gut microbes when the supply of exogenous glycans is limited.^16^

Besides consuming diet- and host-derived glycans, bacteria can also produce glycans themselves (Figure 2). The most common bacterium-derived glycans are lipopolysaccharides, which are produced by Gram-negative bacteria.^30^ In addition, peptidoglycan, exopolysaccharides, capsular polysaccharides, wall polysaccharides and teichoic acids are microbial glycans synthesized intracellularly and transported to the extracellular surface.^31^ These polymers play vital roles in the modulation of gut bacterial colonization, immune responses and pathogenesis,^32^ but they can also serve as a reservoir of glycans for the gut microbiota.

Box: Impact of glycan metabolism on host health. Exogenous glycans, either animal/plant-based or HMOs, are usually considered prebiotics due to their health-promoting properties. The chemical structures of animal-derived glycans (both *N*- and O-glycans) and HMOs are very similar to the glycans present in the intestinal mucus layer. Hence, when supplied in sufficient amounts, bacteria inhabiting the intestinal mucus layer are likely to degrade and utilize these diet-derived polysaccharides rather than utilizing glycans of mucin. Diet-derived glycans thereby contribute to maintenance of the integrity of the intestinal barrier.^16^Plant-derived glycans (mostly dietary fibers) are fermented by the gut microbiota into SCFAs, which have well documented beneficial characteristics, such as facilitating the growth of *Bifidobacterium*, improving glucose tolerance, reducing inflammatory responses and restoring colonic barrier function.^13^ However, Singh et al. reported that, although dietary fibers did ameliorate metabolic syndrome by reducing adiposity and improving glycemic control in multiple strains of dysbiotic mice, microbial fermentation of soluble fibers (but not insoluble fibers) induced cholestasis and hepatocellular carcinoma (HCC) in certain contexts.^17^ For instance, prolonged feeding of inulin-enriched compositionally defined diets to mice resulted in development of cholestatic HCC. In contrast, no indications of liver disease were observed when mice consumed a grain-based rodent chow supplemented similar amounts of inulin.^17^ This was presumably caused by the production of large quantities of butyrate, which exceeded the host’s tolerance threshold. As high butyrate levels have been shown to be toxic for hepatocytes and colonocytes *in vitro* and *in vivo*, they may have aggravated the hepatic and colonic inflammation and disrupted the enterohepatic circulation.^18,19^ In a murine colorectal cancer model, intake of dietary insoluble fibers was reported to promote tumorigenesis in a dose- and time-dependent manner, and this was associated with increased levels of butyrate in feces and bile acids in serum.^20^ These results suggest that dietary fibers and their metabolites (SCFAs) may have health-promoting effects, but that they may also be associated with certain risks, largely depending on the dietary conditions.^21^ Therefore, promoting “high fiber” intake, especially to individuals with dysbiosis and those who consume high quantities of processed foods, should be done with caution and be evaluated systematically, taking potential long-term adverse effects into account.^20,22,23^

## A significant number of glycans contain amino sugars as their crucial and integral component

Amino sugars are a class of carbohydrates in which one or more hydroxyl groups are substituted by amino groups. These compounds are widely found as constituents in carbohydrate chains of glycoproteins and glycolipids and are considered to be physiologically and pathologically important. Amino sugars can make up a significant portion of the carbohydrate component of glycans. Although certain foods contain amino sugars or their precursors, glycans are considered to be the major source of amino sugars in the intestine. For instance, animal-derived glycosaminoglycans are linear polysaccharides whose structural units (disaccharide) consist of an amino sugar (either N-acetyl-glucosamine (GlcNAc) or GalNAc and another carbohydrate.^33^ To date, more than 60 amino sugars are known. The most important and most abundant amino sugars include glucosamine, galactosamine, and their acetylated forms GlcNAc and GalNAc. While microbial glycan metabolism has been extensively studied,^6^ the metabolism of amino sugars by microbes in the human gut has not been systematically investigated.

The actual concentration range of GalNAc in the human intestine is still unclear. More than 100 O-linked oligosaccharides were identified in human sigmoid colon biopsy sample, most of which were based on the core 3 structure (GlcNAcβ1-3GalNAc) (Figure 1e).^34^ Additionally, monosaccharides have been quantified in bovine small intestinal contents. This revealed a total carbohydrate concentration of 4.2 mm, including 0.72 mm GalNAc and 0.89 mm GlcNAc.^35^ In line with this, mucin isolated from the calf small intestine contained 47.4% carbohydrates by weight, with GalNAc and GlcNAc accounting for 24.1% and 23.6% of carbohydrates, respectively.^36^ These findings suggest that large quantities of GalNAc are present within the intestinal lumen and mucus, where they can be accessed and utilized by bacteria.

## Blood-type antigens as a major source of GalNAc through bacterial hydrolysis

A recent study addressed the GalNAc relative abundance per gram of dried cecal content in a mosaic pig population and reported a strong positive correlation between GalNAc concentration and the A blood-type of the pigs, suggesting that the A blood-type antigen is a major source of GalNAc in the intestine.^37^ Pigs have an AO blood group system that is comparable to the human ABO blood group system. In humans, the ABO gene encodes a glycosyltransferase that determines an individual’s blood-type by modifying the immunodominant terminal oligosaccharides in glycolipids and glycoproteins on the surface of red blood cells (RBCs), thereby constituting either A-, B- or O-blood-type antigens. The A-allele of the ABO gene encodes a GalNAc transferase, resulting in a blood-type antigen that terminates with GalNAc (known as A-blood-type antigen). Cell-free blood-type antigens can be secreted into the intestinal mucus layer in individuals who possessing a *FUT2* gene encoding an active fucosyltransferase 2 enzyme that allows bodily secretion of blood-type antigens into saliva, mucus and the gastrointestinal tract. Similarly, the presence of GalNAc pathways in the human gut microbiome was strongly associated with A blood-type in FUT2 secretors,^38^ confirming blood-type-relevant antigens as the major source of mucosal GalNAc also in humans.

## Bacterial-expressed glycoside hydrolases cleave GalNAc from glycans

**Figure 3.**
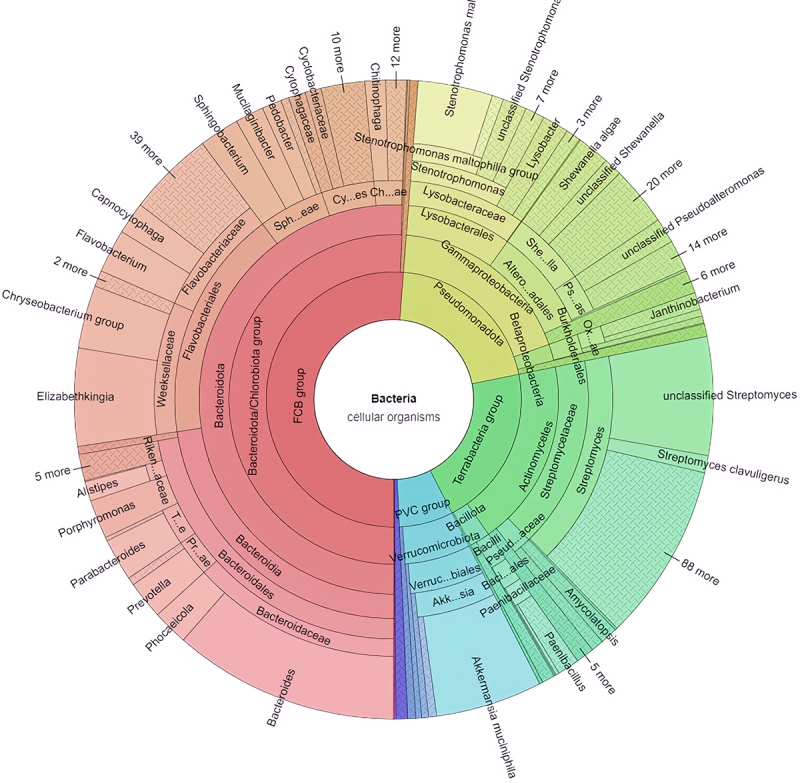
The GH109 family is broadly expressed in bacteria. The krona plot shows the GH109 family identified in the taxonomy of bacteria. The inner to outer circles represent the domain to genus levels. The colors depict different taxonomies. In the CAZy database, 1722 genomes from bacteria harbor the GH109 genes. For more details, refer to the CAZy database (http://www.cazy.org/IMG/krona/GH109_krona.html).

To enable gut microbial utilization of antigen-linked GalNAc, it first needs to be cleaved from blood-type antigens or other glycans by bacterial glycoside hydrolases (GHs). The GHs are a large family of enzymes that catalyze the hydrolysis of glycosidic bonds of complex carbohydrates. Different GH family members can conduct different hydrolysis reactions. The GH 27/36/109 families encode exo-α-N-acetyl-galactosaminidases (exo-α-GalNAcase), which can cleave the α-1,3-linkage between GalNAc and galactose in the A-antigen and release GalNAc into the intestinal lumen.^38^ The linkage between the GalNAc moieties and Ser/Thr parts of mucin can be cleaved by endo-α-N-acetyl-galactosaminidases (endo-α-GalNAcase, GH101/129 families).^39,40^ Further release of GalNAc by cleaving the α/β linkage from carbohydrate chains can be performed by exo-α-GalNAcase (GH27/36/109 families) and β-N-hexosaminidase (GH3/20 families).^41^ GH families are widely present and redundant in bacterial communities.^42^ For example, the GH109 family genes can be detected in more than 10,000 bacterial genomes, including those of common gut microbes like *Faecalibacterium prausnitzii*, *Collinsella aerofaciens* and *Akkermansia muciniphila* (Figure 3). Interestingly, use of bacterial enzymatic activity to cleave the glycoside bond may have great potential in clinical application. Bacterial GH families were recently found to be able to target and remove the carbohydrate extensions of A- and B-antigens to generate universal O blood-type, which could have a great impact on improving transfusion compatibility.^43^ While the efficiency of *A. muciniphila* GH109 for cleaving GalNAc from A-type antigens is rather low, GH110 enzymes (α-galactosidase) can efficiently cleave galactose from B-type antigens, thereby dampening the immune response by about 80% upon crossmatching of these GH110-treated B-type RBCs with A and AB plasma. Further dampening of the immune response (>90%) was achieved when B-type RBCs were incubated with a combination of GH110 and GH20 (β-N-Acetylgalactosaminidase).^43^

## The GalNAc utilization pathway in bacteria

**Figure 4.**
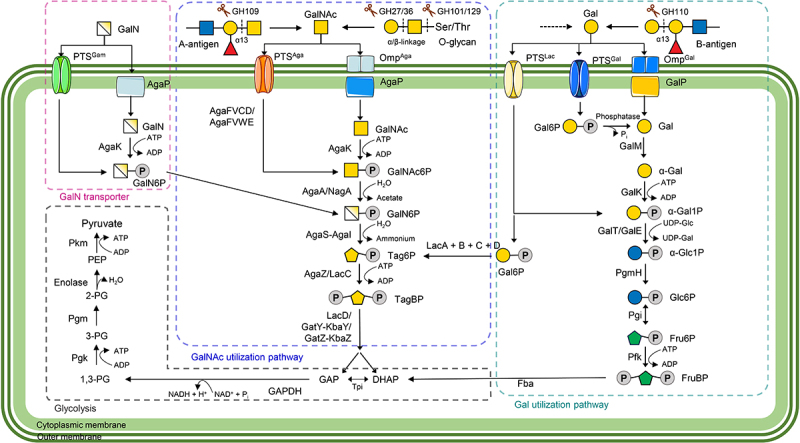
Schematic overview of GalNAc utilization pathways. The diagram shows four different pathways, highlighted by dashed lines colored pink (GalN), medium blue (GalNac), dark cyan (gal) and light gray (Glycolysis). Abbreviations of the genes/enzymes in each pathway are listed in alphabetical order in Table 1. GalN, Galactosamine (or Gam); PTS, phosphotransferase system; GalN6P, glucosamine-6-phosphate; GalNAc, N-acetyl-galactosamine (or Aga); GalNAc6P, GalNAc-6-phosphate; GH, glycoside hydrolase; Tag6P, tagatose-6-phosphate; TagBP, tagatose-1,6-bisphosphate; Lac, Lactose; gal, Galactose; Gal1P: galactose-1-phosphate; Gal6P, galactose-6-phosphate; glc, Glucose; Glc1P, glucose-1-phosphate; Glc6P, glucose-6-phosphate; fru, Fructose; Fru6P, fructose-6-phosphate; FruBP, fructose-1,6-biphosphate; GAP, glyceraldehyde-3-phosphate; DHAP, dihydroxyacetone phosphate; GAPDH, glyceraldehyde-3-phosphate dehydrogenase; 1,3-PG, 1,3-Bisphosphoglycerate; 3-PG, 3-Phosphoglycerate; 2-PG, 2-Phosphoglycerate; PEP, Phosphoenolpyruvate.

When GalNAc is cleaved and released, it can be taken up and metabolized by gut microbes. The GalNAc utilization pathway comprises five steps, including transmembrane transport and intracellular utilization (Table 1 and Figure 4), as we outline below.

**Table 1.** List of genes involved in the GalNAc and galactose utilization pathways.

GalNAc utilization pathway		Gal utilization pathway	
Abbrev.	Full name (function)	Abbrev.	Full name (function)
AgaA	N-acetylgalactosamine-6-phosphate deacetylase	Fba	Fructose-bisphosphate aldolase
AgaC	Aga/Gam specific phosphotransferase transporter subunit IIC or IIC^Aga^/^Gam^	GalE	UDP-galactose-4-epimerase
AgaD	Aga/Gam specific phosphotransferase transporter subunit IID or IID^Aga^/^Gam^	GalK	Galactokinase
AgaE	Aga specific phosphotransferase transporter subunit IID or IID^Aga^	GalM	Galactose mutarotase
AgaF	Aga/Gam specific phosphotransferase transporter subunit IIA or IIA^Aga^/^Gam^	GalP	Galactose: H(+) symporter
AgaI	Galactosamine-6-phosphate isomerase	GalT	Galactose-1-phosphate uridylyltransferase
AgaV	Aga specific phosphotransferase transporter subunit IIB or IIB^Aga^	Pfk	Phosphofructokinase
AgaK	Aga kinase	Pgi	Phosphoglucose isomerase
AgaP	Aga permease	PgmH	α-phosphoglucomutase
AgaS	galactosamine-6-phosphate deaminase/isomerase	**GalN transporter**	
AgaW	Aga specific phosphotransferase transporter subunit IIC or IIC^Aga^	Abbrev.	Full name (function)
GatY	Tagatose-1,6-bisphosphate aldolase 2	AgaP	Aga permease
GatZ	Tagatose-bisphosphate aldolase subunit	AgaK	Aga kinase
KbaY	Tagatose-1,6-bisphosphate aldolase 1	**Glycolysis**	
KbaZ	Tagatose 6-phosphate aldolase subunit KbaZ	Abbrev.	Full name (function)
LacC	Tagatose-6-phosphate kinase	GAPDH	Glyceraldehyde-3-phosphate dehydrogenase
LacD	Tagatose-bisphosphate aldolase	Enolase	Phosphopyruvate hydratase
NagA	N-acetylglucosamine-6-phosphate deacetylase	Pgk	Phosphoglycerate kinase
Omp	Outer membrane protein	Pgm	Phosphoglycerate mutase
PTS	Phosphotransferase system	Pkm	Pyruvate kinase
		Tpi	Triose phosphate isomerase

### Step 1. Transmembrane transport and phosphorylation of GalNAc

GalNAc is taken up into the bacterial cytoplasm by the GalNAc-specific phosphotransferase system (PTS, also known as AgaPTS), which is encoded by different gene clusters. This transport system differs between bacterial species. For instance, in *F. prausnitzii*, four genes – *agaF*, *agaV*, *agaC* and *agaD* – encode proteins that respectively serve as the functional sub-domains IIA, IIB, IIC and IID of the AgaPTS system II complex.^38,44^ However, in *Escherichia coli*, the sub-domains IIC and IID of AgaPTS are encoded by the *agaW* and *agaE* genes, respectively, as the *agaC* and *agaD* genes in *E. coli* code for the functional domains IIC and IID, respectively, of the GamPTS system. Additionally, functional domain IIA (encoded by the *agaF* gene) is shared by the AgaPTS and GamPTS systems in *E. coli* (Figure 4).^44^ In *Shewanella* species, which lack an AgaPTS system, the GalNAc-outer membrane transporter (AgaOMP) and GalNAc permease and kinase (AgaP and AgaK) system substitutes for the AgaPTS system.^45^

### Step 2. Hydrolysis of GalNAc-6-phosphate (GalNAc6P)

GalNAc deacetylase (AgaA) catalyzes the hydrolysis of the N-acetyl group of GalNAc6P to produce D-galactosamine 6-phosphate (GalN6P) and release an acetate molecule.^46^ However, *agaA* is not the only gene involved in this step. This process is not affected after deleting the *agaA* gene in *E. coli*, as these bacteria possess the *nagA* gene (encoding N-acetyl-glucosamine-6-phosphate deacetylase), which substitutes for AgaA in this process.^47^

### Step 3. Deamination-isomerization of GalN6P

The bi-functional enzyme AgaS (GalN6P deaminase/isomerase, encoded by *agaS*) catalyzes the conversion of GalN6P into D-tagatose-6-phosphate (Tag6P) and an ammonium ion via deamination-isomerization. While the function of GalN6P deaminase/isomerase was previously assigned to the AgaI enzyme,^46^ it was later confirmed that AgaI plays an auxiliary role in this step.^45^ Part of the substrate of AgaS enzymes can also be derived from the GalN (galactosamine) transporter pathway (Figure 4).

### Step 4. Phosphorylation of Tag6P

The gene *lacC* encodes Tag6P kinase (LacC), which facilitates the formation of D-tagatose 1,6-bisphosphate (TagBP) from Tag6P through an energy-dependent process. In addition, AgaZ (TagBP aldolase 1, also known as KbaZ) has been proposed to function as a Tag6P kinase.^45^ Part of Tag6P may also be derived from the Gal (galactose) utilization pathway (Figure 4), as reviewed in detail by Kuiper et al.^48^ and Reece et al.^49^

### Step 5. Cleavage of TagBP

Tagatose-bisphosphate aldolase (TBPA), encoded by the *lacD* gene, catalyzes the cleavage of TagBP into dihydroxyacetone phosphate (DHAP) and D-glyceraldehyde 3-phosphate (GAP). In bacteria like *F. prausnitzii*, the catalytic subunits 1 and 2 of TBPA are encoded by the gene clusters *kbaY-gatY* or *gatZ-kbaZ*, respectively. The three-carbon product, GAP, is further converted into pyruvate through a sequence of enzymatic reactions, releasing energy for bacterial use.

## GalNac utilization in clinical applications

GalNAc conceivably represents an important carbon source for various gut microbes. Deletion of the gene encoding GalNAc transferase, which determines AO blood group in pigs, markedly decreased the cecal concentration of GalNAc and thereby reduced the abundance of the GalNAc-utilizing Erysipelotrichaceae species in the porcine gut.^37^ Addition of GalNAc to the culture medium promotes the growth of Erysipelotrichaceae strains,^50,51^ whose abundance has been associated with human disease conditions, including gastrointestinal inflammation and metabolic disorders.^52^ *In vitro* bacterial culture experiments have further confirmed that GalNAc can serve as the sole carbon source to support the growth of bacteria that possess an active GalNAc utilization pathway, including *F. prausnitzii*, one of the most abundant bacteria in the human gut.^38^ This bacterium was recently found to promote human health, while depletion of *F. prausnitzii* has been reported in multiple diseases, including cardiometabolic diseases,^53^ mental diseases, cancers, chronic kidney disease,^54^ Alzheimer’s-type dementia,^55^ and intestinal-related disorders.^56–58^ The pivotal role played by *F. prausnitzii* in human health is primarily related to its ability to utilize dietary fibers for SCFA-production and its ability to enhance antitumor immunity.^59^ Furthermore, the products of bacterial GalNAc metabolism, GAP and DHAP, are also important intermediates in the glycolysis pathway that generates pyruvate. The glycolysis pathway plays important roles in cancer, heart failure and neurodegeneration.^60–62^ In addition, bacterial GalNAc utilization genes are generally associated with host’s cardiometabolic health, particularly in individuals who can secret A-antigens (A or AB blood type) into their intestinal tract,^38^ suggesting that controlling the abundance of GalNAc-utilizing, health-beneficial bacteria in the intestine by manipulating the concentration of GalNAc or the intake of GalNAc-containing glycans may therefore hold great potential to improve human health status.^63^ Important to note that the GalNAc pathway is critical for GalNAc utilization but has no effect on bacterial utilization of various monosaccharides, such as glucose, lactose, mannose, and fructose.^38^ Thus, GalNAc-mediated microbial intervention might differ from dietary fiber-mediated interventions, and its specificity could open a new avenue for precision microbial manipulation.

## Summary and future perspectives

As we have shown, the interactions between gut microbiota and amino sugars are complex, yet crucial for host health. Exogenous and endogenous glycans can modulate the structure and function of the gut microbial community by providing community members with their preferred saccharides as a source of energy. The cleavage of glycans by bacterial GH families releases monosaccharides, including the amino sugar GalNAc, from polysaccharides. Evidence from human and pig studies further indicates that endogenous blood-type-related antigens represent the primary GalNAc source, although exogenous glycans also contribute substantially. This knowledge can potentially be exploited to selectively promote the colonization and growth of beneficial bacteria in the intestine by increasing GalNAc levels or by selecting or engineering GalNAc-utilizing strains.^31,56^ Bacteria capable of degrading glycans and utilizing amino sugars also possess numerous genes and enzymes that may present other routes to potential clinical applications. However, more research is needed to specifically determine if and how bacterial amino sugar metabolism modulates human health. Methods to do so include animal models as well as *in vitro* and *in silico* models. For example, gnotobiotic animal models can be used to verify the effects of specific bacteria carrying amino sugar utilization genes on the host’s health status.^64^ Such models may be employed to identify novel therapeutic strategies that harness the power of the gut microbiota to improve human health.
